# SIRT2 promotes base excision repair by transcriptionally activating OGG1 in an ATM/ATR-dependent manner

**DOI:** 10.1093/nar/gkae190

**Published:** 2024-03-30

**Authors:** Anke Geng, Jiahui Sun, Huanyin Tang, Yang Yu, Xiyue Wang, Jingyuan Zhang, Xiaona Wang, Xiaoxiang Sun, Xiaofang Zhou, Neng Gao, Rong Tan, Zhu Xu, Ying Jiang, Zhiyong Mao

**Affiliations:** Shanghai Key Laboratory of Maternal Fetal Medicine, Clinical and Translational Research Center of Shanghai First Maternity and Infant Hospital, Frontier Science Center for Stem Cell Research, School of Life Sciences and Technology, Tongji University, Shanghai 200092, China; Shanghai Key Laboratory of Maternal Fetal Medicine, Clinical and Translational Research Center of Shanghai First Maternity and Infant Hospital, Frontier Science Center for Stem Cell Research, School of Life Sciences and Technology, Tongji University, Shanghai 200092, China; Shanghai Key Laboratory of Maternal Fetal Medicine, Clinical and Translational Research Center of Shanghai First Maternity and Infant Hospital, Frontier Science Center for Stem Cell Research, School of Life Sciences and Technology, Tongji University, Shanghai 200092, China; Shanghai Key Laboratory of Maternal Fetal Medicine, Clinical and Translational Research Center of Shanghai First Maternity and Infant Hospital, Frontier Science Center for Stem Cell Research, School of Life Sciences and Technology, Tongji University, Shanghai 200092, China; School of Medicine, Tongji University, Shanghai 200092, China; Shanghai Key Laboratory of Maternal Fetal Medicine, Clinical and Translational Research Center of Shanghai First Maternity and Infant Hospital, Frontier Science Center for Stem Cell Research, School of Life Sciences and Technology, Tongji University, Shanghai 200092, China; Shanghai Key Laboratory of Maternal Fetal Medicine, Clinical and Translational Research Center of Shanghai First Maternity and Infant Hospital, Frontier Science Center for Stem Cell Research, School of Life Sciences and Technology, Tongji University, Shanghai 200092, China; Shanghai Key Laboratory of Maternal Fetal Medicine, Clinical and Translational Research Center of Shanghai First Maternity and Infant Hospital, Frontier Science Center for Stem Cell Research, School of Life Sciences and Technology, Tongji University, Shanghai 200092, China; Department of Oncology, Xiangya Cancer Center, Xiangya Hospital, Central South University, Changsha 410008, China; Shanghai Key Laboratory of Maternal Fetal Medicine, Clinical and Translational Research Center of Shanghai First Maternity and Infant Hospital, Frontier Science Center for Stem Cell Research, School of Life Sciences and Technology, Tongji University, Shanghai 200092, China; Department of Oncology, Xiangya Cancer Center, Xiangya Hospital, Central South University, Changsha 410008, China; Shanghai Key Laboratory of Maternal Fetal Medicine, Clinical and Translational Research Center of Shanghai First Maternity and Infant Hospital, Frontier Science Center for Stem Cell Research, School of Life Sciences and Technology, Tongji University, Shanghai 200092, China; Shanghai Key Laboratory of Maternal Fetal Medicine, Clinical and Translational Research Center of Shanghai First Maternity and Infant Hospital, Frontier Science Center for Stem Cell Research, School of Life Sciences and Technology, Tongji University, Shanghai 200092, China; Shanghai Key Laboratory of Maternal Fetal Medicine, Clinical and Translational Research Center of Shanghai First Maternity and Infant Hospital, Frontier Science Center for Stem Cell Research, School of Life Sciences and Technology, Tongji University, Shanghai 200092, China; School of Medicine, Tongji University, Shanghai 200092, China; Shanghai Key Laboratory of Signaling and Disease Research, School of Life Sciences and Technology, Tongji University, Shanghai 200092, China

## Abstract

Sirtuin 2 (SIRT2) regulates the maintenance of genome integrity by targeting pathways of DNA damage response and homologous recombination repair. However, whether and how SIRT2 promotes base excision repair (BER) remain to be determined. Here, we found that independent of its catalytic activity SIRT2 interacted with the critical glycosylase OGG1 to promote OGG1 recruitment to its own promoter upon oxidative stress, thereby enhancing OGG1 promoter activity and increasing BER efficiency. Further studies revealed that SIRT2 was phosphorylated on S46 and S53 by ATM/ATR upon oxidative stress, and SIRT2 phosphorylation enhanced the SIRT2-OGG1 interaction and mediated the stimulatory effect of SIRT2 on OGG1 promoter activity. We also characterized 37 cancer-derived SIRT2 mutants and found that 5 exhibited the loss of the stimulatory effects on OGG1 transcription. Together, our data reveal that SIRT2 acts as a tumor suppressor by promoting OGG1 transcription and increasing BER efficiency in an ATM/ATR-dependent manner.

## Graphical Abstract

**Graphical Abstract:**
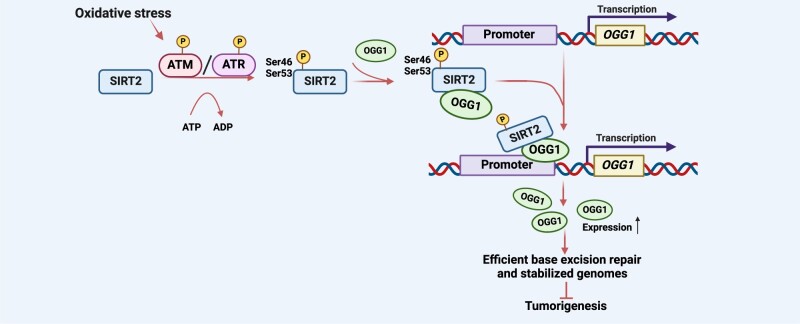

## Introduction

A rise in genomic instability may aberrantly inactivate tumor suppressor genes while activating oncogenes, thereby promoting tumorigenesis (1,2). In response to different kinds of DNA damage, various types of DNA repair pathways have evolved to efficiently eliminate the damage to preserve genomic stability, therefore suppressing tumorigenesis (3,4). Among all types of DNA damage, damage to DNA bases arising from endogenously generated reactive oxygen species (ROS), exogenous chemicals or irradiation occurs very frequently. It is estimated that thousands of DNA base lesions are generated per human cell per day (5). Therefore, efficient base excision repair (BER) is critical to the maintenance of genome integrity. The BER pathway is activated by the removal of the damaged bases by a glycosylase such as OGG1. The resulting abasic sites are further cleaved by the AP endonuclease APE1, generating DNA nicks that need to be processed by a number of DNA end processors, including TDP1/2, PNKP, Aprataxin, APE1 and Pol β. The processed DNA ends with a one-nucleotide gap are filled by Pol β, and this event is followed by XRCC1-Lig3- or Lig1-mediated ligation of the single-strand break (SSB) to complete the BER process (6–8). Although knocking out key BER factors such as Pol β and XRCC1 in mice often leads to embryonic lethality (9,10), cells of numerous different types of cancer tissues harbor mutated Pol β, and several XRCC1 polymorphisms have been associated with breast cancer or lung cancer in Asian patients (11,12). In addition, Pol β expression negatively regulates the progression of breast cancer and lung cancer by promoting the demethylation of the CDH13 promoter (13). Moreover, simultaneously knocking out the two redundant glycosylases, Ogg1 and Myh increases the incidence of tumorigenesis in mice (14).

The ATM (ataxia-telangiectasia mutated) and ATR (ATM- and Rad3-Related) kinases are two of the farthest upstream kinases that respond to a variety of types of DNA damage (15–17). Although most studies on ATM and ATR are related to DNA double-strand breaks (DSBs) or defects in DNA replication progression, accumulating evidence indicates that ATM, ATR or their downstream substrates are involved in regulating oxidative stress-induced DNA damage (18,19). ATM directly participates in BER by phosphorylating TDP1 at Ser81 to promote XRCC1-mediated recruitment of TDP1 to base damage sites (20). CHK2, the downstream kinase of ATM, phosphorylates XRCC1 at Thr284 to facilitate its recruitment to DNA damage sites (21). Although the direct roles of ATR and its downstream kinase CHK1 in the BER pathway remain to be established, the 9–1–1 complex, which is regulated by the ATR–CHK1 axis, promotes BER by targeting several BER factors (22–26).

SIRT2, as a member of the Sirtuin family, participates in the regulation of numerous physiological and pathological processes, including tumorigenesis, age-related neurodegenerative diseases, obesity, aging, dysregulated cell differentiation and homeostasis, infection, inflammation, autophagy, dysregulation of mitosis and genome instability (27–35). As an NAD+-dependent deacetylase, SIRT2 functions to regulate these processes by deacetylating a number of histone and nonhistone substrates, including histone H3, histone H4, CDK9, Myc, SMC1A, RIP1 and so on (34,36–40). SIRT2 is a critical regulator in maintaining genomic stability (41,42). SIRT2 deacetylates BARD1 to enhance the BARD1-BRCA1 interaction, thereby promoting DSB repair by HR (43). In addition, SIRT2 alleviates replication stress by deacetylating CDK9 and ATRIP (38,44). Moreover, although the target protein remains unknown, SIRT2 protects neurons from cisplatin-induced DNA damage that can be repaired via the TC-NER pathway (45). Recent reports have indicated that SIRT2 combats oxidative stress through a number of regulatory mechanisms (46–50), such as deacetylation of FOXO3a (51). However, whether SIRT2 directly participates in regulating the repair of DNA base damage arising from oxidative stress is unknown, and the underlying regulatory mechanisms have not been characterized.

Here, we demonstrated that SIRT2 promotes BER independent of its deacetylase activity. In response to oxidative stress, ATM and ATR phosphorylate SIRT2 at S46 and S53. Phosphorylation of SIRT2 enhances its interaction with the glycosylase OGG1, resulting in the recruitment of OGG1 to its own promoter to increase OGG1 transcription and BER efficiency. We also identified several cancer-associated SIRT2 mutants with loss of the stimulatory effects on OGG1 transcription and BER efficiency, indicating that SIRT2 functions as a tumor suppressor by promoting BER for genome stabilization.

## Materials and methods

### Cell culture

All HCA2-hTERT fibroblasts and HEK293 cells were maintained in Dulbecco's modified Eagle's medium (KeyGEN BioTECH, Cat. # KGM12800) supplemented with 10% (v/v) FBS (Life Technologies, Cat. # 16000), 1% (v/v) penicillin/streptomycin (Life Technologies, Cat. # 11140-050) and 1% MEM Nonessential Amino Acids (Life Technologies, Cat. # 15140-122). All cells were cultured in a 37°C incubator with 5% CO_2_.

### Plasmid transfection and reagents

The ORF of SIRT2 fused to Flag or GFP at the C-terminus was inserted into the backbone of pEGFP-N1. The ORF of OGG1 fused to His or GFP at the C-terminus was inserted into the backbone of pEGFP-N1. Mutants of SIRT2 and OGG1 were generated based on wild-type plasmids.

For HCA2-hTERT fibroblasts, transfection was performed on a Lonza 4D electroporator with the program DT-130. Eukaryotic expression plasmids were introduced into HEK293 cells using the polyethylenimine (PEI) transfection method.

Viral vectors containing shRNAs were generated based on the pLKO.1 vector. The shRNA sequences were as follows: shSIRT2-1, 5′-GCCAACCATCTGTCACTACTT-3′; shSIRT2-2, 5′-GCTAAGCTGGATGAAAGAGAA-3′; shOGG1-1, 5′-GTATGGACACTGACTCAGA-3′; and shOGG1-2, 5′-TACTTCCAGCTAGATGTT-3′. The sequence of OGG1 siRNA was 5′- GAUCAAGUAUGGACACUGA -3′.

The antibodies employed in the study included anti-SIRT2 (Abcam, Cat. # ab211033); anti-OGG1 (Abcam, Cat. # ab124741; ab233214); anti-ATM-S1981p (CST, Cat. # 5883); anti-ATR-T1989p (ABclonal, Cat. # AP1248); anti-P-(Ser/Thr) ATM/ATR substrate (CST, Cat. # 2851); anti-ATR (ABclonal, Cat. # A13951); anti-ATM (CST, Cat. # 2873); anti-acetylated lysine (CST, Cat. # 9441); anti-Lig3 (ABclonal, Cat. # A1887); anti-XRCC1 (Abcam, Cat. # ab134056); anti-PARP1 (CST, Cat. # 9532); anti-Pol β (ABclonal, Cat. # A1681); anti-His (Abmart, Cat. # M20020); anti-Flag (ABclonal, Cat. # AE005); anti-GFP (Abcam, Cat. # ab290) and anti-TUBULIN (ABclonal, Cat. # AC007).

The chemicals employed in the study included potassium bromate (KBrO_3_) (Adamas-beta, Cat. # 82673); ATM Kinase Inhibitor (KU-55933) (Selleck, Cat. # S1092); and ATR Kinase Inhibitor (VE-822) (Selleck, Cat. # S7102).

### FACS analysis

Forty-eight to seventy-two hours post-transfection, cells were harvested and resuspended in 0.2 ml PBS for FACS analysis on a FACSVerse flow cytometer (BD Biosciences, USA). At least 20 000 events were analyzed. All data were subsequently analyzed using FlowJo software.

### Survival assay and FACS analysis of apoptosis

HCA2-hTERT fibroblasts were seeded at a density of 8 × 10^4^ cells per plate. On Day 2 post-seeding, the cells were treated with 20 mM KBrO_3_ for 1 hour and then cultured in drug-free medium for the indicated times. Cells were counted and harvested according to the manufacturer's instructions for the apoptosis assay with an Annexin V-FITC Apoptosis Detection Kit (Beyotime, Cat. # C1062L). Samples were analyzed on a BD Accuri C6 flow cytometer (BD Biosciences, USA).

### Analysis of BER efficiency

The pEGFP-N1 vector was incubated with methylene blue at a concentration of 500 μM, and the mixture was irradiated with visible light at a distance of 18 cm for 3 h. The damaged pEGFP-N1 vector was then purified and cotransfected with a vector encoding DsRed2 into cells. On Day 3 post-transfection, cells were harvested for FACS analysis. The ratio of GFP^+^ cells to DsRed2^+^ cells was used to measure BER efficiency.

### Luciferase assay

HCA2-hTERT fibroblasts were electroporated with the indicated firefly luciferase reporter vectors and pRL-SV40 Renilla vectors prior to a 2-day incubation. On Day 3 post-transfection, cells were collected and subsequently lysed according to the manufacturer's instructions. Relative luciferase activities were measured by a dual-luciferase reporter system (Promega, Cat. # E1910) using a GloMax Luminometer (Promega, Cat. # E5311). Each assay was performed at least three times independently.

### Immunoprecipitation

Cells were harvested, washed once with 1 × PBS, and lysed with lysis buffer (20 mM HEPES, pH 8.0; 0.2 mM EDTA; 5% glycerol; 150 mM NaCl; 1% NP-40) for 15 min on ice. The lysate was then sonicated on ice for 3 min before being centrifuged at 13 500 rpm for 15 min at 4°C. The supernatant was then prepared for immunoprecipitation by two methods. In the first method, the supernatant was pretreated with protein A/G agarose (Abmart, Cat. # A10001M) and IgG (Santa Cruz, #sc-2025) for 1 h at 4°C. After centrifugation, the supernatant was collected and incubated with the indicated antibodies overnight. Afterward, the samples were incubated for 2 h at 4°C after 30 μl of protein A/G agarose was added to the mixture. In the other method, cell lysates were incubated with anti-DYKDDDK magnetic beads (Selleck, Cat. # B26102) or GFP-Trap (ChromoTek, Cat. # gta-100) overnight at 4°C. All precipitated samples were washed three times with lysis buffer and boiled for 10 min with 2 × sample buffer (1% SDS; 10 mM EDTA; 50 mM Tris–HCl, pH 8.1; and protease inhibitors). Whole-cell lysates were used as input as a control.

### ChIP assay

HEK293 cells were transfected with 5 μg of the indicated plasmids and cultured for 24 h before being treated with 40 mM KBrO_3_. Thirty minutes after KBrO_3_ treatment, cells were harvested for the ChIP assay as previously described (76). After precipitation, chromatin and input fragments were used as templates for real-time PCR with the following primers: 5′-CGGCTCTCGGAGAACGGGCTG-3′ and 5′-GTTCTCCAGGGGCAGACCACA-3′.

### Alkaline comet assay

Cells were seeded at a density of 2 × 10^4^ cells/well in 6-well plates. On Day 3, cells were treated with KBrO_3_ at a concentration of 40 mM for 0.5 h or with 100 μM H_2_O_2_ for 4 h. Then, the cells were collected, resuspended in PBS and diluted to 3 × 10^5^ cells per ml before the comet assay was performed. The detailed procedure is described in the manufacturer's instructions (Trevigen, Cat. # 4250-050-K). For formamidopyrimmidine DNA glycosylase (FPG) treatment, on Day 2 post seeding, cells were treated with KBrO_3_ at a concentration of 15 mM for 0.5 h, and then recovered in free-drug medium for indicated time. After lysis, the slides were washed 3 times with enzyme reaction buffer (40 mM HEPES, 0.1 M KCl, 0.5 mM EDTA, 0.2 mg/ml BSA, pH 8.0 with KOH), and then incubated with FPG in a 1:5000 dilution with the enzyme buffer at 37°C for 1 h. Tail moments were used to quantify the amount of DNA damage using CometScore software (casplab_1.2.3b2).

### Quantitative RT−PCR

Total RNA was extracted using a commercial kit (TIANGEN, Cat. # DP419) and reverse transcribed into cDNA by a HiScript III 1st Strand cDNA Synthesis Kit (Vazyme, Cat. # R312-02). Real-time quantitative PCR was performed with FastStart DNA Master SYBR Green Mix (Roche, 4913914001) on a ViiA 7 Real-Time PCR system (Applied Biosystems). The average threshold cycle (Ct) of quadruplicate reactions was determined, and expression was analyzed by the ΔΔCt method. The relative expression levels were normalized to the level of GAPDH. The primers used to amplify OGG1 were as follows: OGG1-mRNA-F-1, 5′-CACACTGGAGTGGTGTACTAGC-3′; OGG1-mRNA-R-1, 5′-CCAGGGTAACATCTAGCTGGAA-3′; OGG1-mRNA-F-2, 5′-ACTCCCACTTCCAAGAGGTG-3′; OGG1-mRNA-R-2, 5′-GGATGAGCCGAGGTCCAAAAG-3′.

## Results

### SIRT2 increases BER efficiency

**Figure 1.**
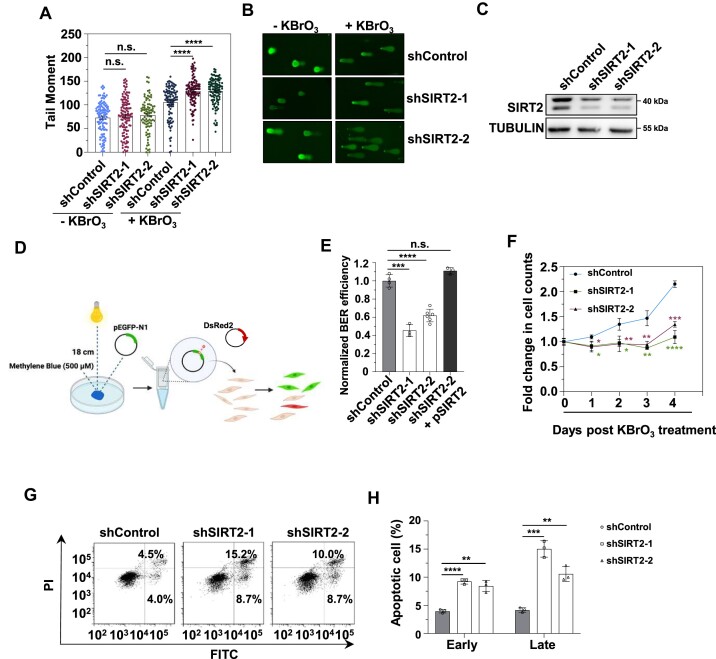
SIRT2 promotes BER for genome stabilization. (**A**) The analysis of genomic instability in control and SIRT2-depleted HCA2-hTERT cells in the presence of the oxidative stress inducer KBrO_3_ using the comet assay. The tail moment was employed as the measure of genomic instability, and at least 100 cells were analyzed using the software CometScore. The error bars indicate the s.e.m. values. The Mann–Whitney *U* test was used for statistical analysis. ****P < 0.0001; n.s., not significant. (**B**) Representative images of the comet assay. (**C**) Western blot analysis of SIRT2 expression in control and SIRT2-depleted HCA2-hTERT fibroblasts. (**D**) Schematic depiction of the experimental principle of BER efficiency analysis. (**E**) Analysis of BER efficiency in the indicated HCA2-hTERT cells. The error bars indicate the S.D. values. Student's *t* test was used for statistical analysis. ***P < 0.001; ****P < 0.0001; n.s., not significant. (**F**) The effect of depleting SIRT2 on cell proliferation. HCA2-hTERT cells were treated with KBrO_3_ at a concentration of 20 mM for 1 h and maintained in culture for the indicated days. At different time points post-KBrO_3_ treatment, cells were harvested and counted. The error bars indicate the S.D. values. Student's *t* test was used for statistical analysis. *P < 0.05; **P < 0.01; ***P < 0.001; (**G, H**) Analysis of apoptosis rates of control and SIRT2-depleted HCA2-hTERT cells in the presence of KBrO_3_. On Day 3 post-KBrO_3_ treatment, cells were harvested for analysis of apoptosis. Representative FACS traces are shown in (**G**). The cells in the upper right rectangles were considered early apoptotic cells, while those in the lower right rectangles were considered late apoptotic cells. The quantitative data are shown in (**H**).

To test whether SIRT2 participates in the maintenance of genome integrity in the presence of oxidative stress, we first performed a comet assay to analyze the changes in genomic stability in control or SIRT2-depleted HCA2-hTERT cells upon oxidative stress induced by KBrO_3_ or H_2_O_2_. We found that the tail moment, which reflects the degree of genomic instability, was significantly increased in SIRT2-depleted HCA2-hTERT cells in the presence of KBrO_3_ or H_2_O_2_ (Figure 1A–C, Supplementary Figure S1a, b). In contrast, under normal conditions, depleting SIRT2 had either a very weak or nonsignificant effect on genomic instability (Figure 1A, B, Supplementary Figure S1a, b). These data suggest that SIRT2 might be involved in regulating the repair of oxidative stress-induced DNA damage.

Therefore, we manipulated the expression of SIRT2 and analyzed the BER efficiency in HCA2-hTERT cells using our well-established plasmid reactivation assay for quantifying BER efficiency (52). The pEGFP-N1 plasmid was treated with methylene blue together with visible light to induce 8-oxodG damage (53), and the damaged plasmids were transfected into cells together with a plasmid expressing DsRed2 as an internal control for monitoring the difference in the transfection efficiency between experiments. Afterward, the ratio of GFP^+^ cells to DsRed2^+^ cells was employed as a measure of BER efficiency (Figure 1D). We observed an increase of approximately 2-fold in BER efficiency in HCA2-hTERT cells overexpressing SIRT2 (Supplementary Figure S1c-d). Consistent with the results of SIRT2 overexpression, depleting SIRT2 significantly reduced BER efficiency by approximately 2-fold (Figure 1E). Restoration of SIRT2 expression in SIRT2 knockdown cells rescued the decline in BER efficiency (Figure 1E). Consistently, using a formamidopyrimmidine DNA glycosylase (FPG)-modified alkaline comet assay, we observed that the tail moment of control cells was reduced by 36.14% from 1.5 to 3 h post KBrO_3_ treatment, while that of SIRT2 depleted cells was less pronounced with a reduction by 18.56% (shSIRT2-1) or 25.01% (shSIRT2-2) (Supplementary Figure S1e, f), further confirming that SIRT2 participates in the repair of KBrO_3_-induced DNA base damage.

Then, we analyzed how SIRT2 affects cell proliferation upon oxidative stress. We found that depleting SIRT2 significantly reduced the proliferation of HCA2-hTERT cells in the presence of KBrO_3_ (Figure 1F). We also examined how SIRT2 affected the apoptosis rate in HCA2-hTERT cells upon oxidative stress. We found that depleting SIRT2 significantly increased oxidative stress-induced early apoptosis by ∼2-fold and late apoptosis by ∼2- to 3-fold (Figure 1G, H).

Together, these results suggest that SIRT2 increases BER efficiency for genome stabilization, thereby promoting cell survival and inhibiting apoptosis.

### SIRT2 promotes the expression of OGG1 at the transcriptional level

We first examined whether SIRT2 is present at the oxidative stress-induced DNA damage sites using a well-established killer-red assay (54,55). The assay revealed that SIRT2 is not recruited to DNA damage sites to regulate BER (Supplementary Figure S2).

**Figure 2.**
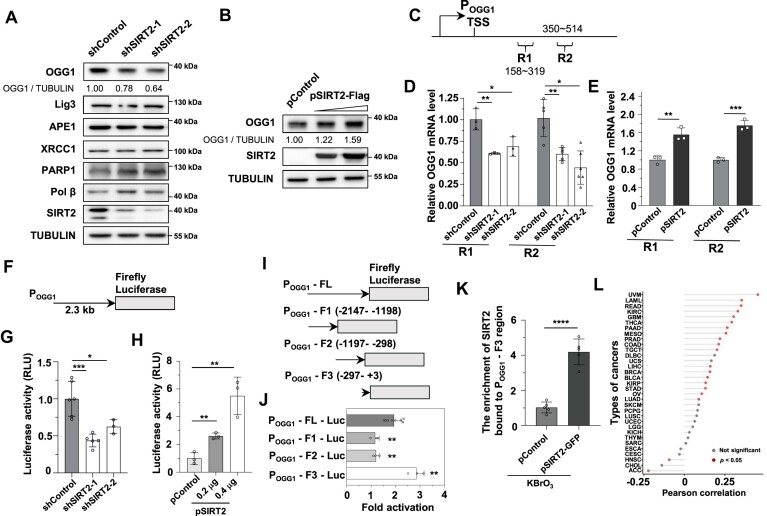
SIRT2 regulates OGG1 expression through transcriptional mechanisms. (**A**) Analysis of the protein expression of different BER factors in control and SIRT2-depleted HEK293 cells. (**B**) Analysis of the protein expression of OGG1 in HEK293 cells transfected with the control vector or the SIRT2 overexpression vector. Quantification of OGG1 protein levels was performed with ImageJ software (A, B). (**C**) The two amplification regions in the OGG1 gene (R1: 158–319; R2: 350–514). (**D**) Analysis of OGG1 mRNA levels in control and SIRT2-depleted HEK293 cells. RNA was extracted for reverse transcription and quantitative PCR with the indicated primers for amplifying the indicated regions in (C). *P < 0.05; **P < 0.01. (**E**) The mRNA level of OGG1 in control and SIRT2-overexpressing HEK293 cells. **P < 0.01; ***P < 0.001. (**F**) Schematic diagram of the firefly luciferase gene driven by the OGG1 promoter (P_OGG1_-Firefly Luciferase). (**G**) Analysis of OGG1 promoter activity in control and SIRT2-depleted HCA2-hTERT cells. The P_OGG1_-firefly luciferase vector was transfected into the cells, and 72 h post-transfection, the cells were lysed for luciferase activity measurement. *P < 0.05; ***P < 0.001. (**H**) Analysis of OGG1 promoter activity in control and SIRT2-overexpressing HCA2-hTERT cells. Cells were transfected with the luciferase reporter vector and either the control vector or a vector encoding SIRT2 at different concentrations. Seventy-two hours post-transfection, cells were lysed for luciferase activity measurement. **P < 0.01. (**I**) Schematic diagrams of the firefly luciferase gene driven by OGG1 promoter fragments. (**J**) Analysis of fold changes in the activity of OGG1 promoter fragments in the presence versus absence of SIRT2 overexpression. **P < 0.01. (**K**) ChIP analysis of the recruitment of SIRT2 to the OGG1 promoter in HEK293 cells. Cells with exogenously introduced control plasmids or pSIRT2-GFP expression vectors were treated with KBrO_3_ and were then lysed and subjected to a ChIP assay with an anti-GFP antibody. ****P < 0.0001. (**L**) The Cleveland dot plot shows the correlation between SIRT2 and OGG1 mRNA expression in 33 different cancer types. The red dot indicates a significant Pearson correlation.

We therefore tested whether SIRT2 affects the expression of important BER factors in SIRT2-depleted cells. We found that knocking down SIRT2 led to a decrease in the protein level of the glycosylase OGG1 but not other key BER factors (Figure 2A). Consistent with this finding, overexpressing SIRT2 increased the protein level of OGG1 in a dose-dependent manner (Figure 2B). Analysis of the OGG1 mRNA level through reverse transcription–quantitative PCR revealed that depleting SIRT2 reduced the mRNA level of OGG1, while overexpressing SIRT2 increased the mRNA level of OGG1 (Figure 2C–E), indicating that the regulation of OGG1 expression by SIRT2 occurs through transcriptional mechanisms. We then cloned a 2.3-kb OGG1 promoter that drives the expression of the firefly luciferase gene (Figure 2F). Using the resulting P_OGG1_-firefly luciferase vector, we found that depleting SIRT2 suppressed OGG1 promoter activity, while overexpressing SIRT2 stimulated OGG1 promoter activity (Figure 2G, H), confirming that SIRT2 regulates OGG1 transcription.

We then separated the OGG1 promoter into three fragments: –2147 to –1198, –1197 to –298 and –297 to +3. By overexpressing SIRT2, we analyzed the changes in the activity of the promoter fragments. We found that SIRT2 retained its stimulatory effect on OGG1 promoter activity only when the –297 to +3 region was intact, whereas the stimulatory effect of SIRT2 was abolished with the other two fragments (Figure 2I, J). Further chromatin immunoprecipitation (ChIP) assays revealed that SIRT2 was recruited to the OGG1 promoter at the –297 to +3 region in response to oxidative stress (Figure 2K).

Importantly, data mining revealed that the mRNA level of SIRT2 was positively correlated with the OGG1 mRNA level in 18 of 33 different types of cancers, confirming that SIRT2 promotes OGG1 expression at the transcriptional level (Figure 2L).

Taken together, these data indicate that SIRT2 is recruited to the OGG1 promoter to increase its transcriptional activity, thereby promoting OGG1 expression and the repair of DNA base damage.

### SIRT2 physically interacts with the OGG1 protein

**Figure 3.**
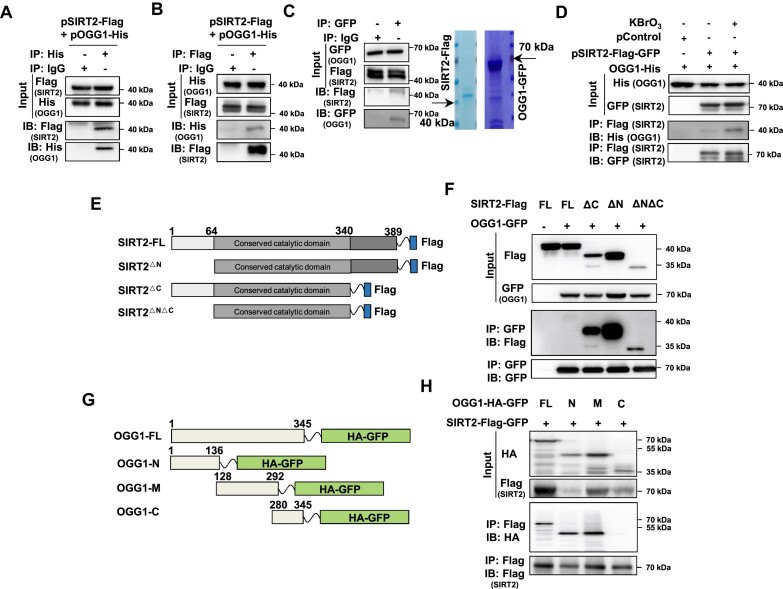
SIRT2 physically interacts with OGG1. (**A, B**) Co-IP analysis of the SIRT2-OGG1 interaction. HEK293 cells were transfected with vectors encoding SIRT2-Flag and OGG1-His. Cells were lysed for co-IP with an anti-His antibody (**A**) or an anti-Flag antibody (**B**) followed by Western blot analysis. (**C**) *In vitro* analysis of the SIRT2-OGG1 interaction. The purified recombinant SIRT2-Flag and OGG1-GFP were coincubated *in vitro* prior to co-IP with an anti-GFP antibody. (**D**) Co-IP analysis of the SIRT2-OGG1 interaction in HEK293 cells treated with KBrO_3_. HEK293 cells transfected with vectors encoding SIRT2-Flag-GFP and OGG1-His were treated with 40 mM KBrO_3_ for 30 min before being lysed for co-IP with Flag beads followed by Western blot analysis. (**E, F**) Co-IP analysis of the interaction between OGG1 and SIRT2 mutant fragments. The SIRT2 mutant fragments are shown in (**E**). Cells were transfected with vectors encoding these mutants tagged with Flag and OGG1 tagged with GFP and were lysed for co-IP with GFP-Trap followed by Western blot analysis (**F**). (**G, H**) Co-IP analysis of the interaction between SIRT2 and OGG1 mutant fragments. The OGG1 mutant fragments are shown in (**G**). Cells were transfected with vectors encoding these mutants tagged with HA and GFP, and SIRT2 tagged with Flag and GFP, followed by the lysis of cells for co-IP with Flag beads and Western blot analysis (**H**).

Since SIRT2 and other members of the Sirtuin family participate in DNA repair by directly interacting with and modifying repair factors, we proposed that SIRT2 might regulate BER by targeting important BER factors. A vector encoding Flag-tagged SIRT2 was transfected into HEK293 cells, and co-IP experiments were then performed with beads coated with an antibody against Flag. The data indicated that OGG1 but no other core BER factors interacted with SIRT2 (Supplementary Figure S3a). Then, we performed co-IP experiments with an antibody against the His tag using lysates of cells transfected with vectors expressing His-tagged OGG1 and Flag-tagged SIRT2. The results demonstrated that OGG1 interacted with SIRT2 in cells (Figure 3A). The reciprocal co-IP experiment further confirmed this conclusion (Figure 3B). We also performed *in vitro* co-IP experiments with purified recombinant SIRT2 and OGG1 proteins. The data revealed that SIRT2 and OGG1 interacts directly (Figure 3C).

Then, we determined whether the SIRT2-OGG1 interaction is enhanced upon oxidative stress. To this end, we treated cells with KBrO_3_ or H_2_O_2_ to induce oxidative stress and then performed co-IP experiments. We found that the interaction between SIRT2 and OGG1 was enhanced in response to KBrO_3_ or H_2_O_2_ (Figure 3D, Supplementary Figure S3b), suggesting that the two factors function cooperatively to promote the repair of oxidative stress-induced DNA damage.

Furthermore, we performed co-IP experiments to determine which domains of the two factors interact with the other protein. We found that removing either the N-terminal or C-terminal domain or removing both the N- and C-terminal domains of SIRT2 did not abolish its interaction with OGG1 (Figure 3E, F), suggesting that the central catalytic domain of SIRT2 is responsible for its interaction with OGG1. We also observed that the N-terminal and central domains but not the C-terminal domain of OGG1 interacted with SIRT2 (Figure 3G, H).

### SIRT2 facilitates the recruitment of OGG1 to its own promoter to increase OGG1 transcription

**Figure 4.**
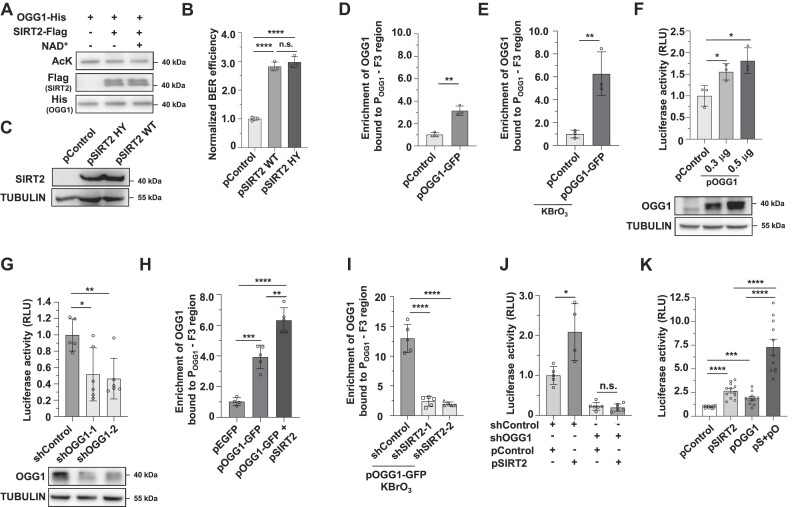
SIRT2 facilitates the recruitment of OGG1 to its own promoter to enhance OGG1 transcription. (**A**) *In vitro* analysis of the acetylation level of OGG1 in the presence of SIRT2. Purified recombinant OGG1 and SIRT2 from HEK293 cells were incubated with each other in the absence or presence of NAD^+^ prior to Western blot analysis with the indicated antibodies. (**B**) Analysis of BER efficiency in HCA2-hTERT cells overexpressing SIRT2 WT or the enzymatically dead H187Y mutant. ****P < 0.0001; n.s., not significant. (**C**) Western blot analysis of the expression of SIRT2 WT or the enzymatically dead H187Y mutant in HCA2-hTERT cells. (**D, E**) ChIP analysis of the recruitment of OGG1 to its promoter in HEK293 cells. Cells with exogenous introduction of the control plasmid or pOGG1-GFP expression vector were left untreated (**D**) or treated with KBrO_3_ (**E**), followed by lysis and a ChIP assay with GFP-Trap. **P < 0.01. (**F**) Analysis of OGG1 promoter activity in control and OGG1-overexpressing HCA2-hTERT cells. Cells were transfected with the luciferase reporter vector and a control vector or a vector encoding OGG1 at different concentrations. Seventy-two hours post-transfection, cells were lysed for luciferase activity measurement. The Western blot image shows the analysis of OGG1 expression in HEK293 cells transfected with increasing amounts of the OGG1 vector. *P < 0.05. (**G**) Analysis of OGG1 promoter activity in control and OGG1-depleted HCA2-hTERT cells. *P < 0.05; **P < 0.01. (**H**) ChIP analysis of the recruitment of OGG1 to its promoter in HEK293 cells transfected with the control or SIRT2 plasmid and the OGG1-GFP plasmid. Cells were transfected with the indicated plasmids and were then lysed and subjected to a ChIP assay with GFP-Trap. **P < 0.01; ***P < 0.001; ****P < 0.0001. (**I**) ChIP analysis of the recruitment of OGG1 to its promoter in control and SIRT2-depleted cells transfected with OGG1-GFP plasmids. ****P < 0.0001. (**J**) Analysis of OGG1 promoter activity in control and OGG1-depleted HCA2-hTERT cells in the presence or absence of SIRT2 overexpression. *P < 0.05; n.s., not significant. (**K**) Analysis of OGG1 promoter activity in HCA2-hTERT cells transfected with vectors encoding OGG1 and/or SIRT2. ***P < 0.001; ****P < 0.0001.

We hypothesized that SIRT2 might deacetylate OGG1 to regulate BER. To test this hypothesis, we performed an *in vitro* deacetylation assay using purified recombinant SIRT2 and OGG1 proteins. We found that SIRT2 did not deacetylate OGG1 *in vitro* (Figure 4A). Further co-IP experiments in SIRT2-depleted cells revealed that SIRT2 depletion did not change the acetylation level of OGG1 (Supplementary Figure S4a). These data indicate that the regulation of BER by SIRT2 probably does not occur through deacetylation of OGG1. Further analysis of BER efficiency using vectors encoding the enzymatically dead SIRT2 H187Y mutant indicated that SIRT2 promoted BER efficiency independent of its enzymatic activity (Figure 4B, C).

In addition to its function in the BER pathway, OGG1 has been reported to function as an important transcription modulator and bind to specialized promoter structures (56). Since SIRT2 promotes BER efficiency independent of its enzymatic activity and its interaction with OGG1 is enhanced by oxidative stress, we proposed that SIRT2 might facilitate the recruitment of OGG1 to its own promoter to stimulate its expression in response to oxidative stress.

We therefore first examined whether OGG1 is recruited to its own promoter to enhance its own transcriptional activity. The ChIP assay revealed that OGG1 was recruited to its promoter at the –297 to +3 region, and oxidative stress further stimulated its recruitment to its own promoter (Figure 4D, E). In addition, we found that overexpression of OGG1 stimulated the activity of its own promoter in a dose-dependent manner (Figure 4F) and that depletion of OGG1 impaired its promoter activity (Figure 4G). Further luciferase assay revealed that the –197 to +3 region in OGG1 promoter is required for OGG1-mediated activation of its own promoter (Supplementary Figure S4b, c). By analyzing the OGG1 promoter activity in cells overexpressing OGG1 catalytically dead mutants OGG1 K249A or D268N (57,58), we also demonstrated that OGG1 stimulated its promoter activity independent of its canonical enzymatic activity (Supplementary Figure S4d). A previous report indicates that OGG1 might cooperate with transcription factors including TFIID, SP1 and NF-κB to regulate transcription of different genes (56), so we further examined whether OGG1 stimulates its own promoter activity through these transcription factors. The luciferase assay revealed that depleting OGG1 diminished the stimulatory effect of SP1 and TFIID on OGG1 promoter activity (Supplementary Figure S4e, f), indicating the two transcription factors are involved in the regulation of OGG1 promoter by OGG1 itself.

Next, we aimed to understand whether SIRT2 interacts with OGG1 to promote the recruitment of OGG1 to its own promoter, thereby stimulating its expression and BER efficiency. The ChIP assay revealed that overexpressing SIRT2 promoted the recruitment of OGG1 to its own promoter (Figure 4H), while knocking down SIRT2 suppressed this recruitment in response to KBrO_3_-induced oxidative stress (Figure 4I). Further *in vitro* Electrophoretic Mobility Shift Assay (EMSA) revealed that OGG1 but not SIRT2 directly bound to its own promoter (–197 to +3), while supplementing SIRT2 greatly enhanced the binding of OGG1 to its own promoter (Supplementary Figure S4g–i).

Consistent with this finding, depleting endogenous OGG1 reduced its promoter activity and completely abolished the stimulatory effect of SIRT2 on OGG1 promoter activity (Figure 4J). ChIP assay also revealed that knocking down endogenous OGG1 reduced the recruitment of SIRT2 to OGG1 promoter (Supplementary Figure S4j). Simultaneously overexpressing SIRT2 and OGG1 synergistically increased OGG1 promoter activity by ∼7.5-fold, while overexpressing SIRT2 or OGG1 alone increased OGG1 promoter activity by ∼2.5-fold or ∼2.0-fold, respectively (Figure 4K).

These data demonstrate that SIRT2 probably forms a complex with OGG1 to stimulate the recruitment of OGG1 to its own promoter, thereby enhancing its promoter activity to increase OGG1 expression. After OGG1 binds to the promoter, the SIRT2-OGG1 complex might fall apart and SIRT2 might quickly fall off the OGG1-DNA complex.

### The promotion of BER by SIRT2 is dependent on the ATM and ATR kinases

**Figure 5.**
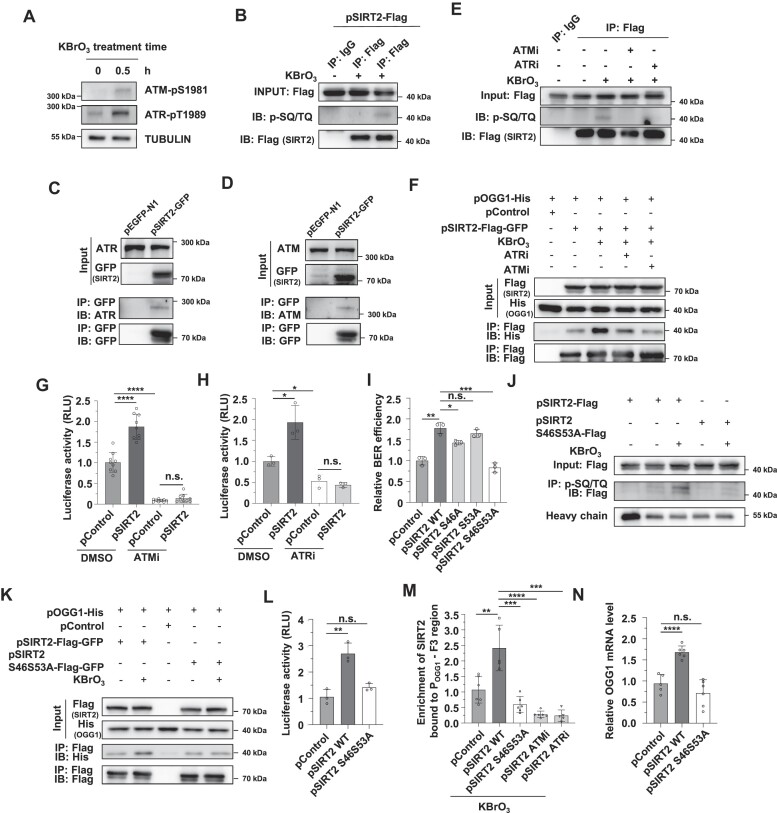
SIRT2 regulates OGG1 transcription in an ATM/ATR-dependent manner. (**A**) Analysis of ATM or ATR phosphorylation in cells treated with KBrO_3_. HEK293 cells treated with 40 mM KBrO_3_ were harvested for protein extraction followed by Western blot analysis with indicated antibodies. (**B**) Analysis of SIRT2 phosphorylation in KBrO3-treated cells. HEK293 cells transfected with SIRT2-Flag plasmids were treated with or without KBrO_3_ and lysed for co-IP with an anti-Flag antibody followed by Western blot analysis with indicated antibodies. **(C, D)** Co-IP analysis of the interaction between SIRT2 and ATM (**C**) or ATR (**D**). (**E**) The effect of ATM or ATR inhibitors on SIRT2 phosphorylation in cells treated with KBrO_3_. KBrO_3_-treated HEK293 cells were incubated with an ATM inhibitor (15 μM) or an ATR inhibitor (10 μM) for 4 hours and were then lysed for co-IP with an anti-Flag antibody followed by Western blot analysis with the indicated antibodies. (**F**) Co-IP analysis of the interaction between SIRT2 and OGG1 in cells treated with ATM or ATR inhibitors. (**G, H**) Analysis of OGG1 promoter activity in HCA2-hTERT cells with or without SIRT2 overexpression in the presence or absence of the ATM inhibitor (**G**) or ATR inhibitor (**H**). *P < 0.05; ****P < 0.0001; n.s., not significant. (**I**) The analysis of BER efficiency in HCA2-hTERT cells transfected with vectors encoding SIRT2 WT, S46A mutant, S53A mutant or S46AS53A mutant. * *P* < 0.05; **P < 0.01; ***P < 0.001; n.s., not significant. (**J**) Analysis of SIRT2 WT and S46AS53A mutant phosphorylation. HEK293 cells transfected with Flag-tagged WT or mutant SIRT2 were treated with or without KBrO_3_ and then lysed for co-IP with an anti-p-(S/T)Q antibody followed by Western blot analysis. (**K**) Co-IP analysis of the interaction between OGG1 and SIRT2 WT or SIRT2 S46AS53A. (**L**) Analysis of OGG1 promoter activity in HCA2-hTERT cells transfected with vectors encoding SIRT2 WT or SIRT2 S46AS53A. **P < 0.01; n.s., not significant. (**M**) ChIP analysis of the recruitment of SIRT2 to OGG1 promoter in cells transfected with the indicated plasmids or subjected to the indicated treatment. **P < 0.01; ***P < 0.001;****P < 0.0001. (**N**) Analysis of the OGG1 mRNA level in cells transfected with vectors expressing SIRT2 WT or the S46AS53A mutant. ****P < 0.0001; n.s., not significant.

Since the SIRT2-OGG1 interaction was enhanced under oxidative stress (Figure 3D, Supplementary Figure S3b), we proposed that SIRT2 is regulated by DNA damage response factors such as the ATM and ATR kinases. However, ATM and ATR are primarily involved in regulating DSB repair; thus, we first examined whether they are activated by oxidative stress. We found that treating cells with KBrO_3_ caused upregulation of ATM or ATR phosphorylation (Figure 5A), indicating that the two kinases also participate in the regulation of the oxidative stress-induced DNA damage response. The conserved motif that ATM and ATR recognize and phosphorylate is the SQ/TQ cluster (59). To test whether SIRT2 is phosphorylated by ATM/ATR upon oxidative stress, we performed co-IP experiments followed by Western blot analysis using a widely used antibody against phosphorylated SQ/TQ. The results revealed that in response to DNA damage induced by KBrO_3,_ SIRT2 was phosphorylated (Figure 5B). Further co-IP experiments revealed that SIRT2 interacted with ATM or ATR (Figure 5C, D). Pretreating cells with ATM or ATR inhibitors abolished the oxidative stress-induced phosphorylation of SIRT2 (Figure 5E). Moreover, inhibiting ATM or ATR kinase activity with their specific inhibitors attenuated the stress-induced enhancement of the SIRT2-OGG1 interaction (Figure 5F) and abolished the stimulatory effect of SIRT2 on OGG1 promoter activity (Figure 5G, H).

By analyzing the amino acid sequence of SIRT2, we predicted that the amino acid residues Ser46 and Ser53 were two potential sites that could be phosphorylated by ATM and ATR, and we constructed vectors expressing SIRT2 S46A, S53A and S46AS53A. We further examined whether the mutations abolished the enhancing effect of SIRT2 on BER efficiency. We found that mutating both of these serine residues into alanine residues abolished the stimulatory effect (Figure 5I, Supplementary Figure S5a), indicating that both residues are critical to the promotion of BER by SIRT2. *In vitro* phosphorylation experiments demonstrated that ATM or ATR may phosphorylate recombinant SIRT2 WT protein but not SIRT2 S46AS53A mutant (Supplementary Figure S5b, c). Further co-IP experiments revealed that the two mutations attenuated the KBrO_3_-induced phosphorylation of SIRT2 (Figure 5J) and abolished the KBrO_3_-induced enhancement of the SIRT2-OGG1 interaction (Figure 5K). Overexpressing the SIRT2 S46AS53A mutant also failed to stimulate OGG1 promoter activity (Figure 5L), promote the recruitment of SIRT2 to OGG1 promoter region (Figure 5M) and increase the OGG1 mRNA level (Figure 5N).

Taken together, our data demonstrate that in response to oxidative stress, ATM and ATR phosphorylate SIRT2 at amino acid residues S46 and S53 to promote its interaction with OGG1, thereby increasing OGG1 transcription and improving BER efficiency.

### Several cancer-associated SIRT2 mutations impair its capacity to promote BER

**Figure 6.**
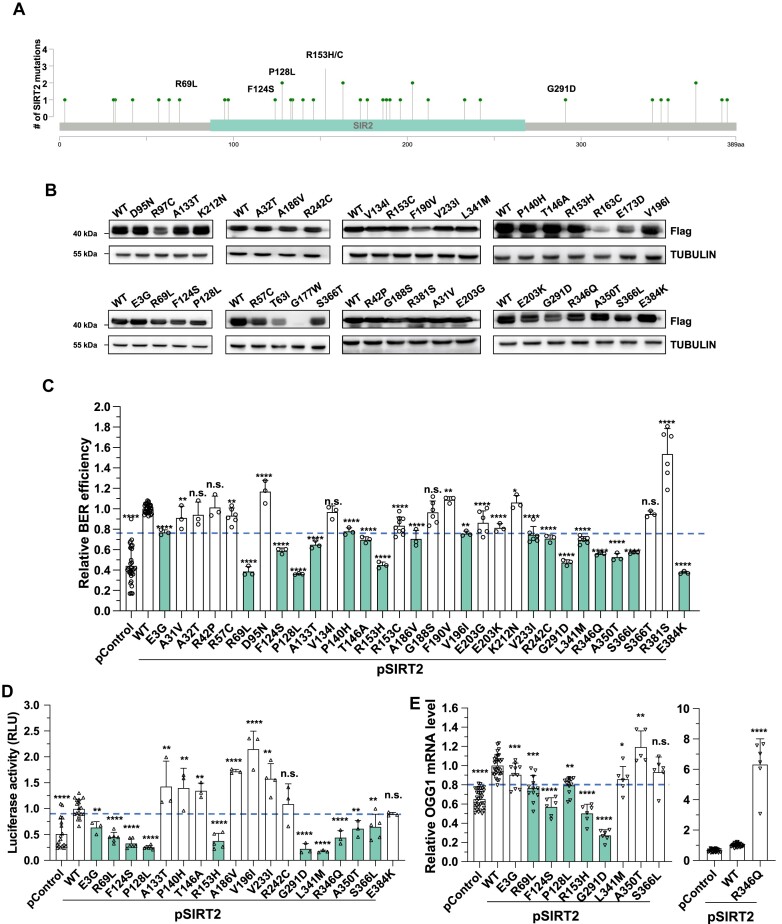
Several cancer-associated SIRT2 mutants do not exert a stimulatory effect on OGG1 transcription and fail to improve BER. (**A**) Lollipop plot of the SIRT2 protein with the alterations present in cancers. (**B**) Western blot analysis of the expression of all SIRT2 mutants in HCA2-hTERT cells. HCA2-hTERT cells were transfected with vectors encoding SIRT2 WT or the indicated 37 mutants at 5 μg. On Day 2 post-transfection, cells were harvested for Western blot analysis. (**C**) Analysis of BER efficiency in HCA2-hTERT cells overexpressing SIRT2 WT or SIRT2 mutants. (**D**) Analysis of OGG1 promoter activity in HCA2-hTERT cells overexpressing SIRT2 WT or SIRT2 mutants. (**E**) Analysis of OGG1 mRNA levels in HEK293 cells overexpressing SIRT2 WT or SIRT mutants.

To determine the functional consequences of SIRT2-mediated regulation of OGG1 transcription, we identified 37 cancer-associated mutations in SIRT2 through data mining using the TCGA database (Figure 6A, Supplementary Figure S6a). We then constructed vectors expressing these SIRT2 mutants. Since cancer-associated mutations often cause rapid protein degradation (60,61) and these mutations apparently affect protein functions, we first examined whether these mutations affect the protein level of SIRT2. We found that 5 of the 37 mutations led to downregulation of the protein expression of SIRT2 (T63I, R97C, R163C, E173D and G177W) (Figure 6B); thus, we excluded these mutants from further analysis.

Using the remaining 32 mutants, we analyzed the efficiency of BER in cells overexpressing these SIRT2 mutants. We found that the stimulatory effect of 18 of the 32 mutants was partially impaired or completely abolished (Figure 6C). The relative BER efficiency in cells overexpressing these mutants was at most 80% of that in cells overexpressing SIRT2 WT. We then further examined whether these SIRT2 mutants affected OGG1 promoter activity. We found that 10 of them failed to enhance or partially lost their stimulatory effects on OGG1 promoter activity (Figure 6D). The relative OGG1 promoter activity in cells overexpressing the 10 mutants was at most 70% of that in cells overexpressing SIRT2 WT. Real-time quantitative PCR analysis further confirmed that the 5 SIRT2 mutants (R69L, F124S, P128L, R153H and G291D) were unable to promote OGG1 mRNA expression as high as SIRT2 WT (Figure 6E). The mRNA level of OGG1 in cells overexpressing the five mutants was less than 80% of that in cells overexpressing SIRT2 WT. Data mining revealed that patients with the five mutations in SIRT2 had high mutation rates across the genomes (Supplementary Figure S6a). Co-IP experiments revealed that three of the five mutants R69L, R153H and G291D could not be phosphorylated in response to oxidative stress (Supplementary Figure S6b). Furthermore, we performed softagar assay to examine whether these mutations affect tumorigenesis (62). Indeed, we found that overexpressing SIRT2 WT reduced the rates of tumorigenesis while all the five mutations abolished the suppressive effect of SIRT2 on tumorigenesis (Supplementary Figure S6c).

Taken together, our data demonstrate that at least five cancer-derived SIRT2 mutations reduce the capability of SIRT2 to promote OGG1 transcription to enhance BER efficiency, thereby contributing to tumorigenesis.

## Discussion

In this report, our data reveal that in response to oxidative stress SIRT2 is phosphorylated at the two serine residues S46 and S53 by ATM/ATR, and the phosphorylated SIRT2 promotes the recruitment of OGG1 to its own promoter to activate its transcription, thereby increasing BER efficiency and preventing tumorigenesis (Supplementary Figure S7).

In the past two decades, numerous targets of SIRT2 have been identified (38,63–65). As an NAD+-dependent deacetylase, SIRT2 deacetylates histone or nonhistone substrates to regulate physiological processes (36–38,63,65,66). SIRT2 is generally considered a tumor suppressor because its expression is downregulated in most cancers, and it restricts cell cycle progression by deacetylating cell cycle-related factors and deacetylating H4K16 to allow chromatin condensation (30,37,41,67–71). Our present study adds another piece of evidence that SIRT2 functions as a tumor suppressor by promoting DNA repair by BER for genome stabilization. However, previous reports also indicated that SIRT2 increases the protein stability of the oncoprotein c-Myc by transcriptionally repressing the expression of the E3 ligase NEDD4 (39), and inhibiting SIRT2 might be a good therapeutic approach in cancer treatment (27,72,73). We propose that the opposite oncogenic and tumor-suppressive roles of SIRT2 might rely on biological contexts such as the type and stage of cancer.

Other Sirtuins, such as SIRT1 and SIRT6, have been reported to function to regulate transcription and DNA repair independent of their enzymatic activities (74,75). To our knowledge, we are the first to demonstrate that SIRT2 plays an important role in DNA repair independent of its enzymatic activity. In addition, this finding also indicates a positive role of SIRT2 in regulating transcription, distinct from its suppressive roles in transcription through targeting histones (36). Our results indicate that beyond targeting the deacetylase activity of SIRT2, developing novel methods to enhance the association of SIRT2 with OGG1 might be a promising approach for cancer prevention and the treatment of neurodegenerative diseases that are associated with the rise in genomic instability resulting from oxidative stress (76).

In addition to functioning in tumorigenesis, SIRT2 is also reported to play important roles in neurodegenerative diseases (77), which are associated with changes in oxidative stress homeostasis (76). Moreover, although it is well established that ATM or ATR mutations may lead to genomic instability, thereby promoting tumorigenesis, patients with ATM or ATR mutations are also reported to have neurodegenerative diseases (78,79). Since ATM and ATR are primarily recognized as the transducers functioning in response to DSBs, while failure to repair DSBs is usually not associated with phenotypes of neurodegeneration, our finding that ATM and ATR regulate BER by modifying SIRT2 may provide a mechanistic explanation for the etiology of neurodegenerative diseases.

Notably, ATM/ATR-SIRT2 axis-mediated recruitment of OGG1 to its own promoter enhances the transcriptional activity of OGG1, which creates a positive feedback loop. Since the phosphorylation of SIRT2 by ATM/ATR and the autophosphorylation of ATM/ATR are the triggers of the positive feedback loop, we speculate that the oxidative stress-induced triggers need to be released after the oxidative stress-induced DNA damage is repaired. There might be different mechanisms to achieve this. For instance, the phosphorylation of SIRT2 could be removed via the action of phosphatases. The phosphatases that are responsible for the dephosphorylation of ATM/ATR have been identified (80,81), but the potential phosphatases that dephosphorylate SIRT2 remain to be determined. In addition, phosphorylated SIRT2 might have a short half-life due to possibly stronger interaction with E3 ligases.

Although our data indicate that SIRT2 regulates BER by increasing the expression of the glycosylase OGG1, which specifically removes 8-oxoG, a number of other glycosylases also participate in the BER pathway by removing different types of damaged bases, such as alkylated bases. Whether SIRT2 regulates the expression of other glycosylases remains to be determined, and the potential underlying mechanisms remain to be identified.

It is of note that both N- and C-terminals of SIRT2 prevent its interaction with OGG1. We speculate that this is a protective mechanism that helps cells to avoid overactivation of DNA repair. Only in the condition of oxidative stress, the occurrence of phosphorylation at the two serine residues in the N-terminal results in increased negative charges and hydrogen-bond networks that might change the structure of SIRT2 to allow stronger interaction with OGG1, thereby stimulating OGG1 transcription to improve DNA repair.

Notably, in this work, we analyzed 37 cancer-associated SIRT2 mutants, and only five of them exhibited loss of the stimulatory effect on OGG1 promoter activity and mRNA expression. Three mutations of them affected the phosphorylation of SIRT2, thereby inhibiting its own promoter activity. For the remaining two mutations, we propose that these mutations might affect other aspects of SIRT2. For instances, the subcellular localization of SIRT2, or the interaction between SIRT2 and OGG1, might be changed by the mutations. However, this warrants further investigation. The other 32 SIRT2 mutants might have gained oncogenic functions, such as promoting the expression of oncogenes, or lost other cancer-preventive functions, such as mediating HR repair and the stabilization of tumor suppressors. Moreover, in the 32 SIRT2 mutants, we observed some inconsistency between BER efficiency and transcriptional activity of OGG1 mediated by SIRT2. This can be explained by that there exists other SIRT2-mediated regulatory mechanisms in BER. Indeed, a previous report indicates that SIRT2 might deacetylate the critical BER factor PARP1, leading to the WWP2-mediated upregulation in ubiqutination and degradation of PARP1(49). These mutations in SIRT2 might change its regulation in PARP1 protein stability and complicate the results in BER efficiency. However, great efforts are still needed to clarify whether and how exactly these mutations affect BER pathway.

## Supplementary Material

gkae190_Supplemental_Files

## Data Availability

The data underlying this article are available in the article and in its online supplementary material.
