# Supplementary material for: SIRT2 promotes base excision repair by transcriptionally activating OGG1 in an ATM/ATR-dependent manner
Source: Nucleic Acids Res. 2024 Mar 30;52(9):5107–20. doi: 10.1093/nar/gkae190 (PMC11109957; doi:10.1093/nar/gkae190)
Supplement: gkae190_Supplemental_Files [file gkae190_supplemental_files.zip › Supplementary Material-R1-V6.docx]

**Supplementary Materials and Methods**

**Oxidative damage induction**

U2OS TRE cells were cultured in 35 mm glass-bottom dishes (MatTek, P35GC-1.5-14-C) at 50-60% confluence 24 h before the transfection. For ROS-induced damage, cells were transfected with plasmids containing KR (TA-KR/tetR-KR) or controls (TA-Cherry/tetR-Cherry) (1). Light-induced KR activation was done by exposing cells to a 15 W cool white fluorescent bulb for 20 min. A Leica DM6500 confocal microscopy was used for live-cell observation and image acquisition. A 594-nm laser was used for bleaching KR.

**Electrophoretic mobility shift assay (EMSA)**

The double-stranded biotin-labled oligonucleotide probes were designed based on the sequences of OGG1 promoter and synthesized by GENEWIZ (Suzhou, China). EMSA was performed using LightShift® Chemiluminescent EMSA Kit (Cat. #20148, Thermo Scientific). Briefly, purified His-tagged OGG1 and Flag-tagged SIRT2 proteins were pre-incubated in the binding buffer at 37 °C for 60 min, followed by mixing with Biotin-labeled probe in a total volume of 20 μL buffer containing 10 mM Tris-HCl (pH 7.5), 5 mM KCl, 1 mM EDTA, 1 mM DTT and 50 ng/μL Poly[d(I-C)]. After that, reaction mixtures were applied to a 6% polyacrylamide gels and electrophoresed in 0.5×TBE buffer.

***In vitro* kinase activity assays**

The purified SIRT2 WT or mutant protein was incubated with ATM or ATR protein in 20 µL reaction buffer containing 50 mM Tris-HCl, pH 7.5, 150 mM NaCl, 5 mM MgCl_2_, 10% glycerol, 2mM ATP and DNA at 30 °C for 90 min. Reactions were terminated by the addition of SDS-PAGE sample loading buffer, followed by Western blot analysis.

**Colony formation in soft agar**

The assay was as previously reported (2,3). Briefly, primary HCA2 fibroblasts stably expressing SV40 large tumour antigen (LT), H-Ras V12 (Ras) and hTERT were electroporated with vectors encoding SIRT2 WT or mutants on a Lonza 4D electroporation machine, followed by hygromycin selection. Cells were split and seeded (1.0×10^5^ cells/well) in a 0.7% noble agar (Sigma) mixed with culture medium (on the top of 1% noble agar with medium) at 37℃. Colonies were photographed and colony numbers were counted in a blinded fashion after 5 weeks.

**Supplementary Figure Legends**

**Supplementary Figure 1.** Related to Figure 1.

**(a)** The analysis of genomic instability in control and SIRT2-depleted HCA2-hTERT cells in the presence of H_2_O_2_ using the comet assay. The tail moment was employed as the measure of genomic instability, and at least 100 cells were analyzed using the software CometScore. The error bars indicate the s.e.m. values. The Mann-Whitney U test was used for statistical analysis. ****P* < 0.001; *****P* < 0.0001; n.s., not significant. **(b)** Representative images of the comet assay. **(c)** Analysis of BER efficiency in the indicated HCA2-hTERT cells. ****P* < 0.001. **(d)** Western blot analysis of SIRT2 overexpression in HCA2-hTERT cells. **(e)** The analysis of genomic instability in control and SIRT2-depleted HCA2-hTERT cells at 1.5 h and 3 h post KBrO_3_ treatment using the FPG-modified alkaline comet assay. The tail moment was employed as the measure of genomic instability, and at least 100 cells were analyzed using the software CometScore. The error bars indicate the s.e.m. values. **(f)** Representative images of the FPG-modified alkaline comet assay.

**Supplementary Figure 2.** Related to Figure 2.

The analysis of localization of BFP-SIRT2 in U2OS TRE cells transfected with KR (TA-KR/tetR-KR) and matched control (TA-Cherry/tetR-Cherry)

**Supplementary Figure 3.** Related to Figure 3.

**(a)** Co-IP analysis of SIRT2 and BER factors. Cells were transfected with SIRT2-Flag expression vectors, and on Day 2, cells were harvested for lysis and co-IP analysis. **(b)** Co-IP analysis of the SIRT2-OGG1 interaction in HEK293 cells treated with H_2_O_2_ at indicated concentrations. HEK293 cells transfected with vectors encoding SIRT2-Flag-GFP and OGG1-His were treated with the indicated DNA damage agent before being lysed for co-IP with Flag beads followed by Western blot analysis.

**Supplementary Figure 4.** Related to Figure 4.

**(a)** Analysis of the acetylation level of OGG1 in HEK293 cells with SIRT2 depletion. Cells transfected with OGG1-HA expression vectors were lysed for co-IP with an anti-HA antibody followed by Western blot analysis with an anti-AcK antibody. **(b)** Schematic diagrams of the firefly luciferase gene driven by OGG1 promoter fragments. **(c)** Analysis of fold changes in the activity of OGG1 promoter fragments in the presence versus absence of OGG1 overexpression. **P* < 0.05; n.s., not significant. **(d)** Analysis of OGG1 promoter activity in HCA2-hTERT cells transfected with control vectors, or plasmids expressing WT OGG1 and enzymatically dead OGG1 mutants. Cells were transfected with the luciferase reporter vector together with the control vector or a vector encoding OGG1 or OGG1 mutants at different concentrations. At 72-hour post-transfection, cells were lysed for luciferase activity measurement. *****P* < 0.0001. **(e)** Analysis of fold changes in the activity of OGG1 promoter fragments in control and OGG1-depleted HCA2-hTERT cells in the presence versus absence of NF-κB, SP1 and TF IID overexpression. ***P* < 0.01; n.s., not significant. **(f)** Western blot analysis of the expression of NF-κB, SP1 and TF IID. **(g-h)** Analysis of the binding affinity of OGG1-His **(g-h)** or SIRT2-Flag **(g)** recombinant protein to biotin-labled OGG1-promoter-F3 oligo by EMSA assay. **(i)** EMSA analysis of binding affinity of OGG1 to biotin-labled OGG1-promoter-F3 oligo in the presensce of recombinant SIRT2-Flag or SIRT2 S46S53A-Flag protein. In terms of SIRT2 amount, twice more SIRT2 in column ++ was added in comparison to that in column +. The asterik in **(g-i)** represents the OGG1-promoter complex. **(j)** ChIP analysis of the recruitment of SIRT2 to OGG1 promoter in control and OGG1-depleted cells transfected with the indicated plasmids or subjected to the indicated treatment. *****P* < 0.0001.

**Supplementary Figure 5.** Related to Figure 5.

**(a)** Western blot analysis of the expression of WT SIRT2 and the indicated phosphorylation mutants. **(b-c)** *In vitro* analysis of the phosphorylation level of SIRT2 in the presence of ATM **(b)** or ATR **(c)**. Purified recombinant SIRT2 or SIRT2S46AS53A was incubated with was incubated with ATM or ATR protein in reaction buffer prior to Western blot analysis with the indicated antibodies.

**Supplementary Figure 6.** Related to Figure 6.

**(a)** The number of non-synonymous mutations among patients presenting SIRT2 mutations across diverse cancer types. **(b)** Analysis of the phosphorylation of SIRT2 WT and mutants in response to KBrO_3_ treatment. HEK293 cells transfected with plasmids expressing Flag-tagged SIRT2 WT or mutants were treated with or without KBrO_3_, and then lysed for co-IP with an anti-p-(S/T)Q antibody followed by Western blot analysis. **(c)** The analysis of colony formation of HCA2 fibroblasts. HCA2 cells stably expressing SV40 large tumour antigen (LT), HRAS V12 (Ras) and TERT were transfected with SIRT2-WT or mutants, followed by plating in soft agar. Colonies were photographed after 5 weeks of growth. Quantification data are shown and represent the mean ± s.e.m. of n = 3 independent experiments. Student’s t test was used for statistical analysis .***P* < 0.01; ****P* < 0.001.

**Supplementary Figure 7.** The model showing that SIRT2 promotes BER by activating OGG1 transcription in an ATM/ATR dependent manner.

Under oxidative stress, ATM and ATR are phosphorylated and activated. The activated ATM/ATR phosphorylates SIRT2 at the two serine residues S46 and S53. The phosphorylated SIRT2 then helps recruit OGG1 to its own promoter to stimulate its own transcription, thereby increasing BER efficiency.

1. Lan, L., Nakajima, S., Wei, L., Sun, L., Hsieh, C.L., Sobol, R.W., Bruchez, M., Van Houten, B., Yasui, A. and Levine, A.S. (2014) Novel method for site-specific induction of oxidative DNA damage reveals differences in recruitment of repair proteins to heterochromatin and euchromatin. *Nucleic Acids Res*, **42**, 2330-2345.

2. Tian, X., Azpurua, J., Hine, C., Vaidya, A., Myakishev-Rempel, M., Ablaeva, J., Mao, Z., Nevo, E., Gorbunova, V. and Seluanov, A. (2013) High-molecular-mass hyaluronan mediates the cancer resistance of the naked mole rat. *Nature*, **499**, 346-349.

3. Liu, H., Zhang, H., Wu, X., Ma, D., Wu, J., Wang, L., Jiang, Y., Fei, Y., Zhu, C., Tan, R. *et al.* (2018) Nuclear cGAS suppresses DNA repair and promotes tumorigenesis. *Nature*, **563**, 131-136.
